# Supplementary material for: Toroidal topology of population activity in grid cells
Source: Nature. 2022 Jan 12;602(7895):123–8. doi: 10.1038/s41586-021-04268-7 (PMC8810387; doi:10.1038/s41586-021-04268-7)
Supplement: Supplementary file 1 — This file contains Supplementary Data; Supplementary Methods and Supplementary References for references cited only in Supplementary Methods. [file 41586_2021_4268_MOESM1_ESM.pdf]

nature portfolio

https://doi.org/10.1038/s41586-021-04268-7

Supplementary information

Toroidal topology of population activity in grid cells

In the format provided by the authors and unedited

nature portfolio

https://doi.org/10.1038/s41586-021-04268-7

Supplementary information

Toroidal topology of population activity in grid cells

In the format provided by the authors and unedited

Supplementary Data

Tuning to coordinates in space and on the inferred torus for all grid cells of all modules (separated into 'pure' and 'conjunctive' categories), except R2 day 2 (Extended Data Fig. 10). Subsets of these plots are shown in Fig. 2b and 4h. Plots from left to right: open-field firing rate map, head-direction tuning curve (black) compared to occupancy of head directions (light gray), temporal autocorrelogram, wagon-wheel firing rate map (depending on recording session), toroidal firing rate maps for OF, WW, REM and SWS (where available)

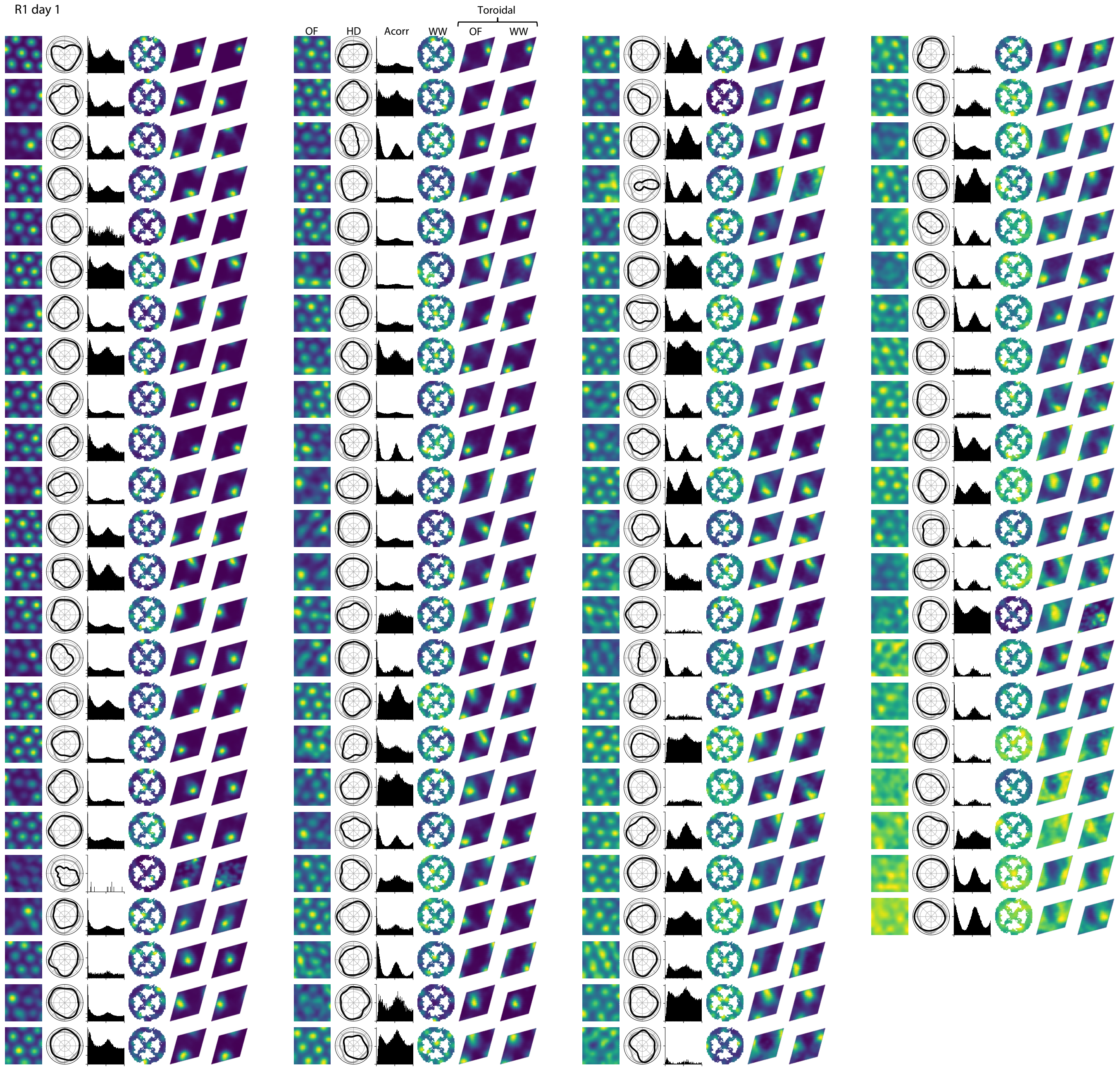
R1 day 1
Toroidal
OF
HD
Acorr
WW
OF
WW

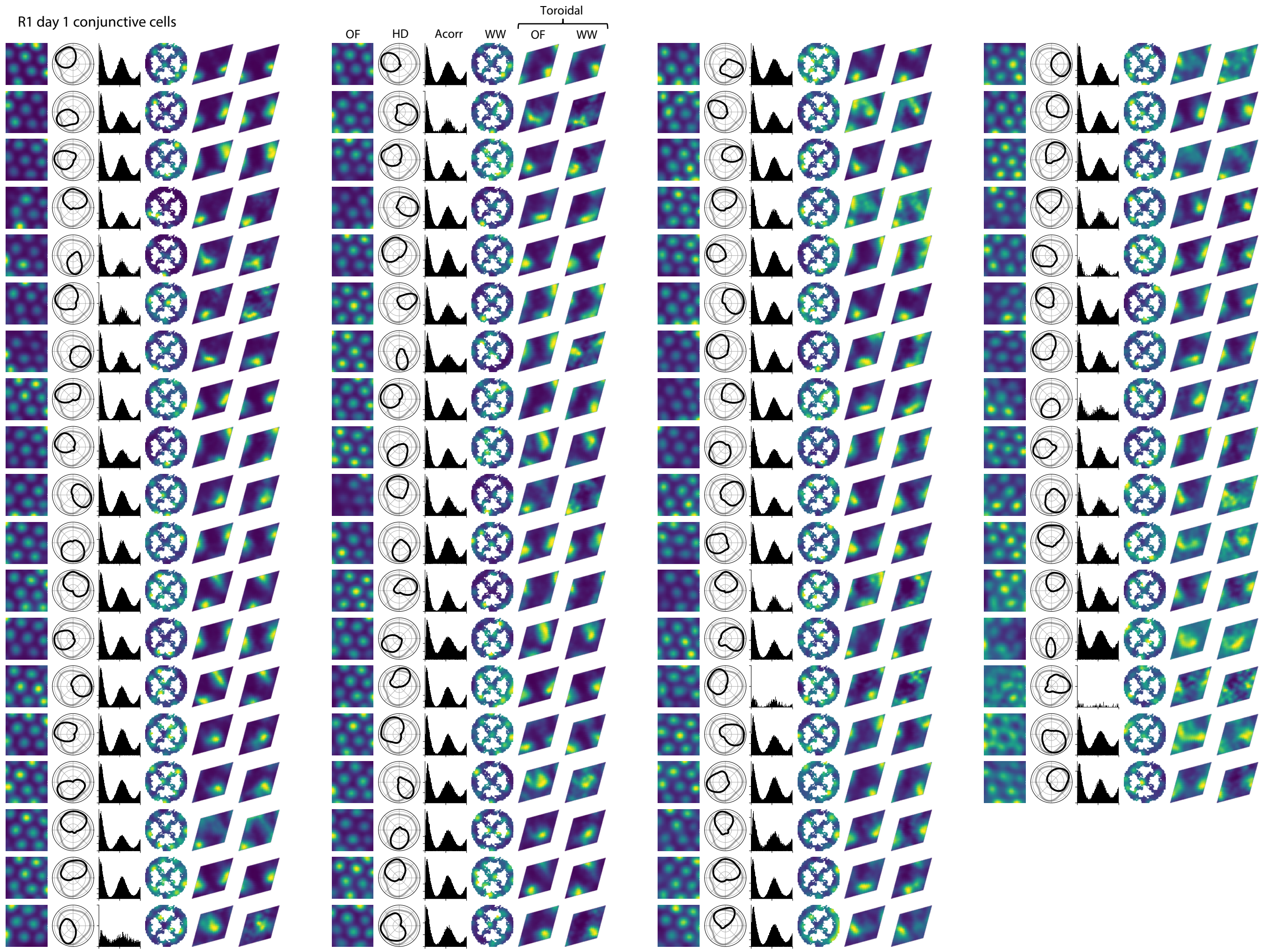
R1 day 1 conjunctive cells
Toroidal
OF
HD
Acorr
WW
OF
WW

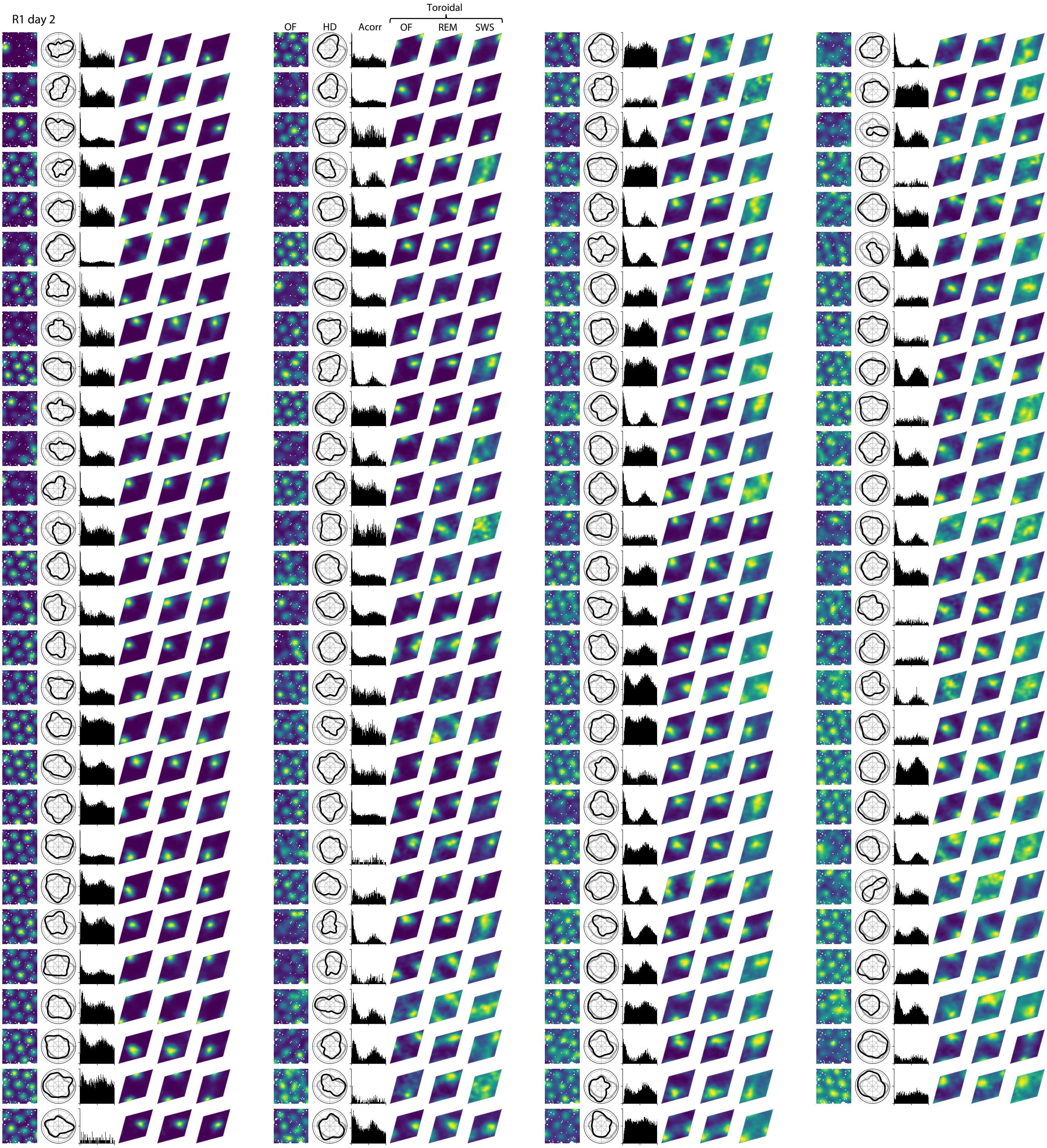

R1 day 2
Toroidal
OF
HD
Acorr
OF
REM
SWS

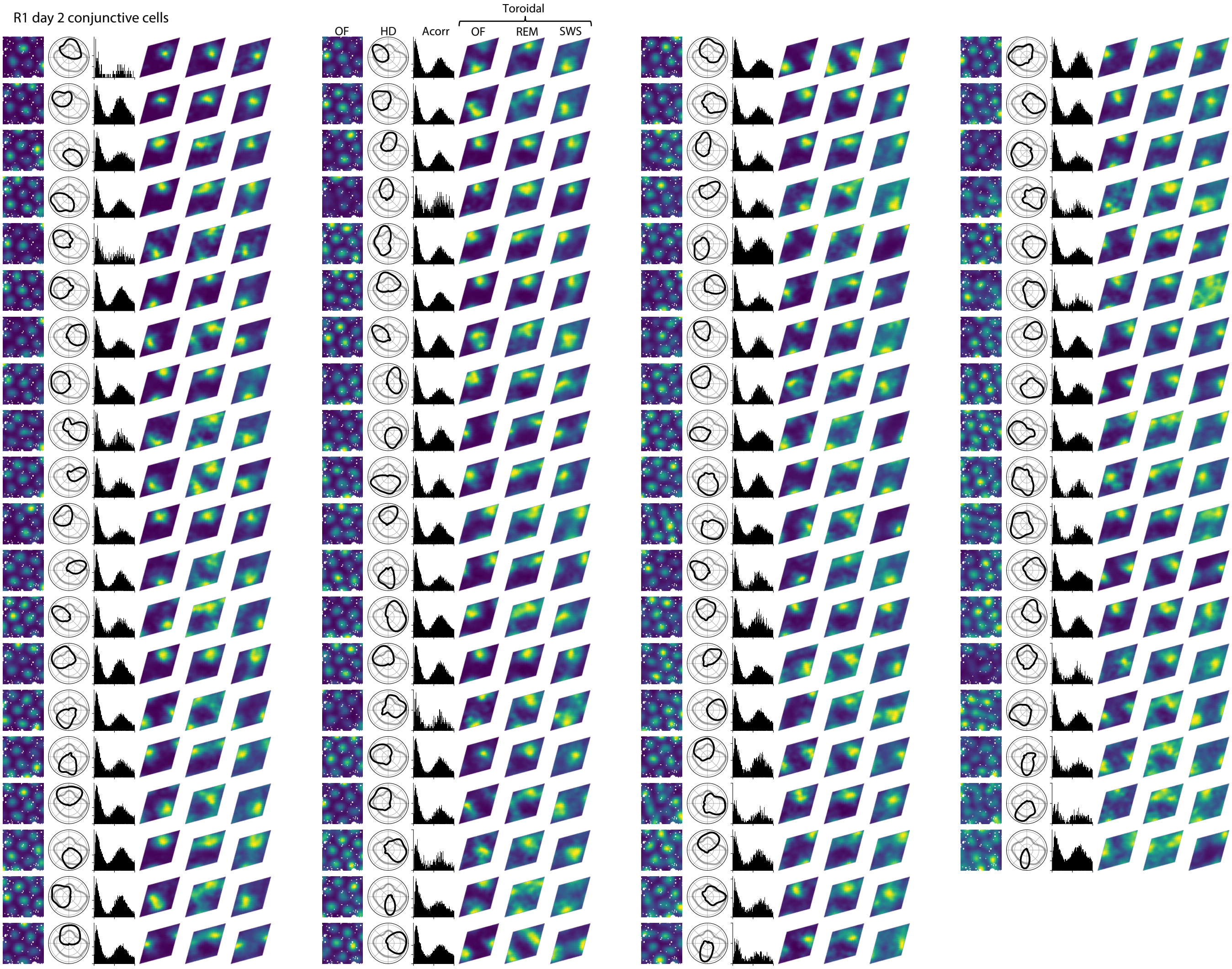
R1 day 2 conjunctive cells
Toroidal
OF
HD
Acorr
OF
REM
SWS

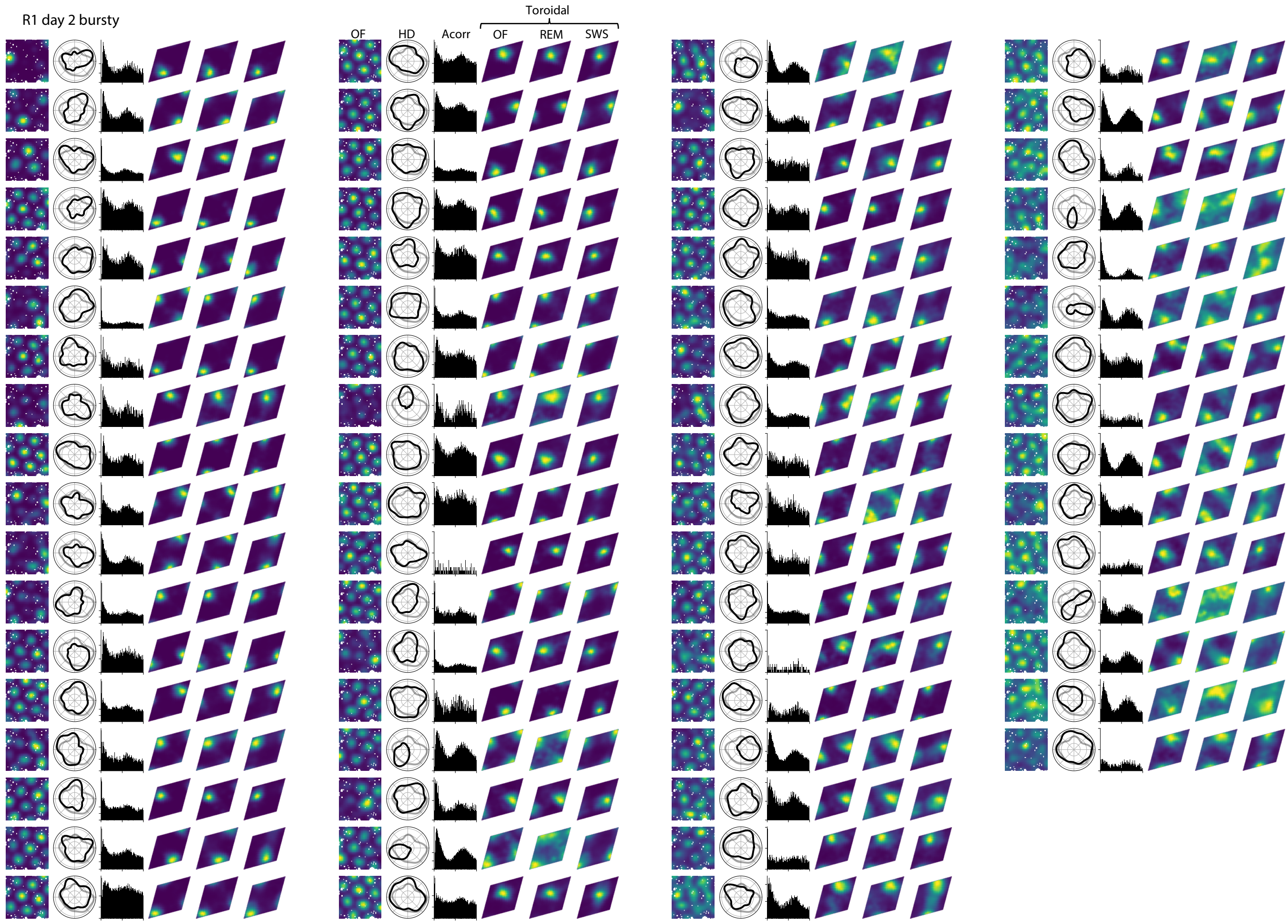
R1 day 2 bursty
Toroidal
OF
HD
Acorr
OF
REM
SWS

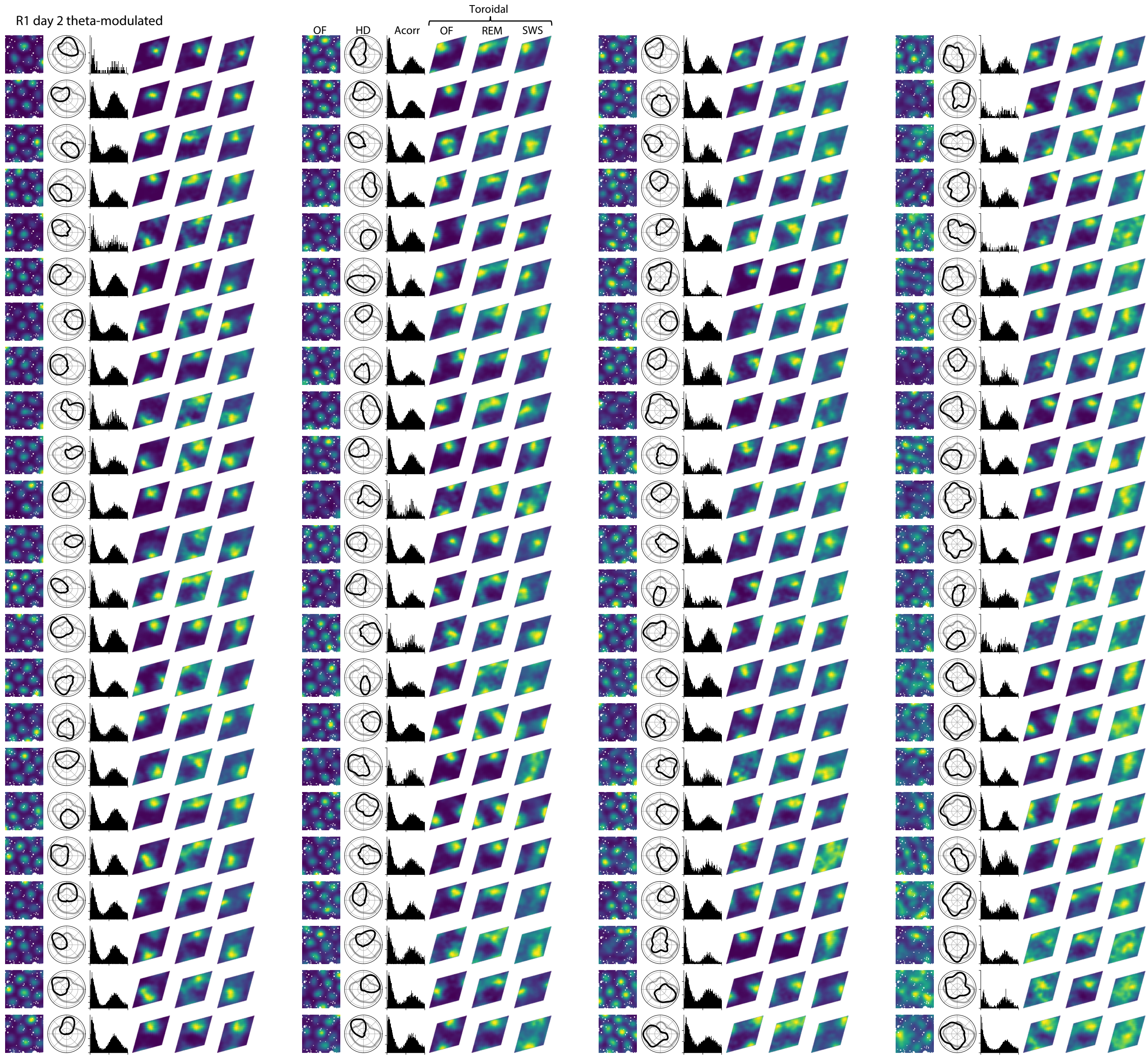
R1 day 2 theta-modulated
Toroidal
OF
HD
Acorr
OF
REM
SWS

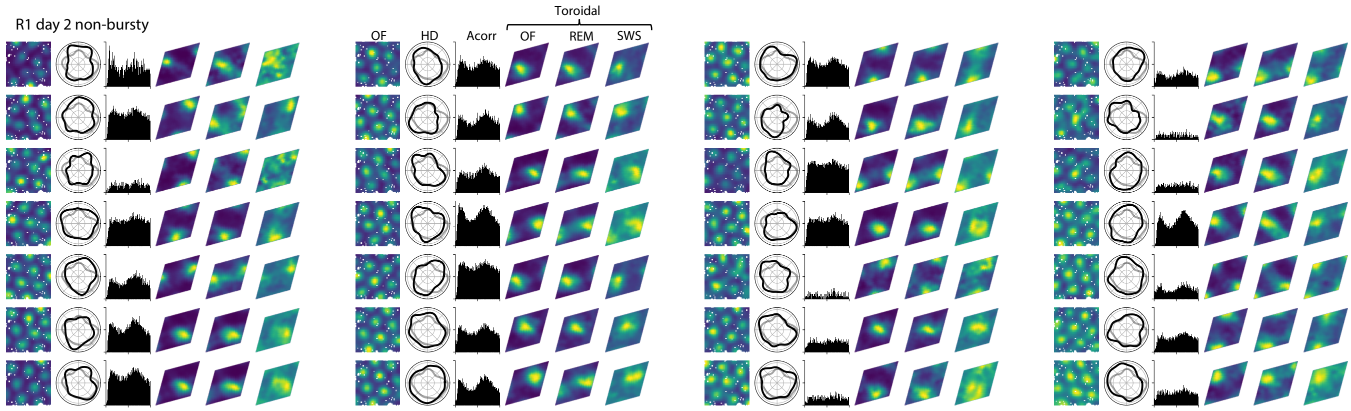
R1 day 2 non-bursty
Toroidal
OF
HD
Acorr
OF
REM
SWS

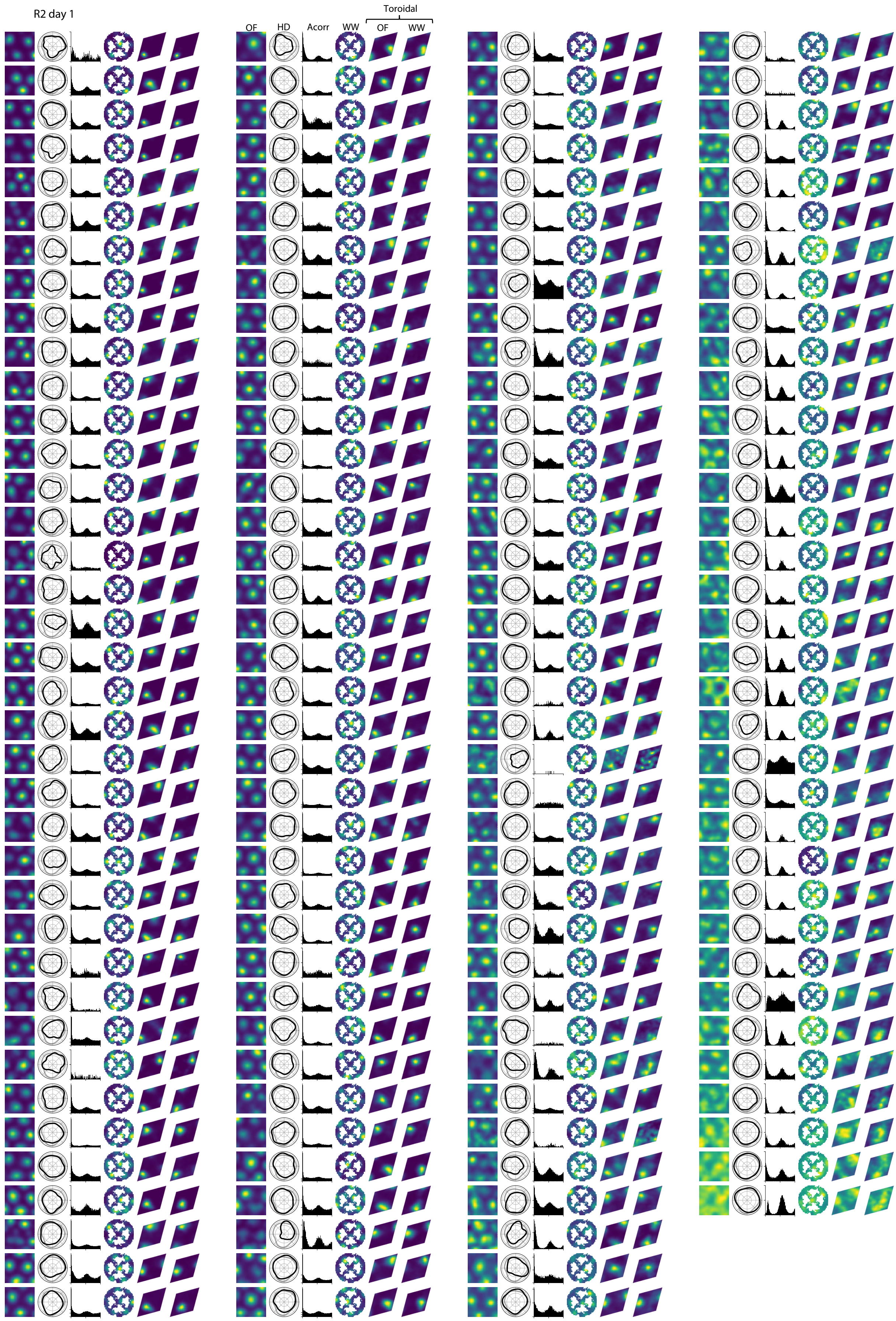
R2 day 1
Toroidal
OF
HD
Acorr
WW
OF
WW

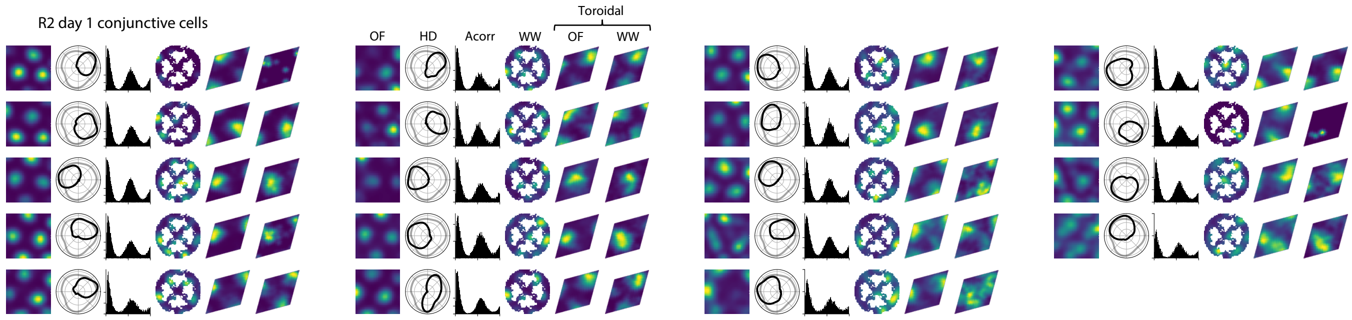

R2 day 1 conjunctive cells
Toroidal
OF
HD
Acorr
WW
OF
WW

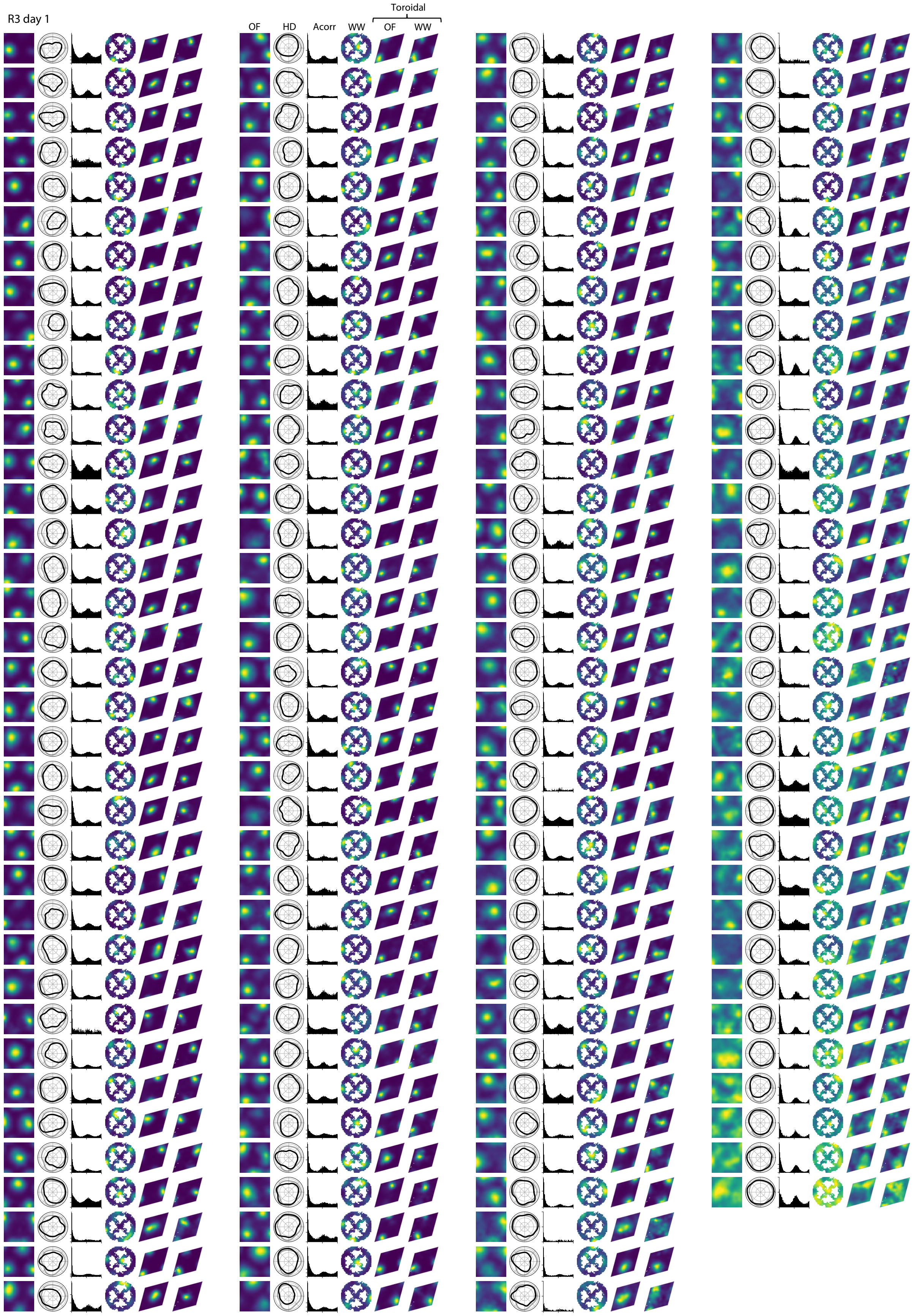
R3 day 1
Toroidal
OF
HD
Acorr
WW
OF
WW

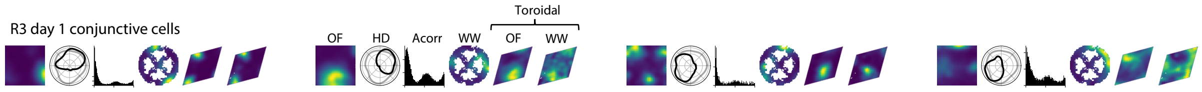
R3 day 1 conjunctive cells
Toroidal
OF
HD
Acorr
WW
OF
WW

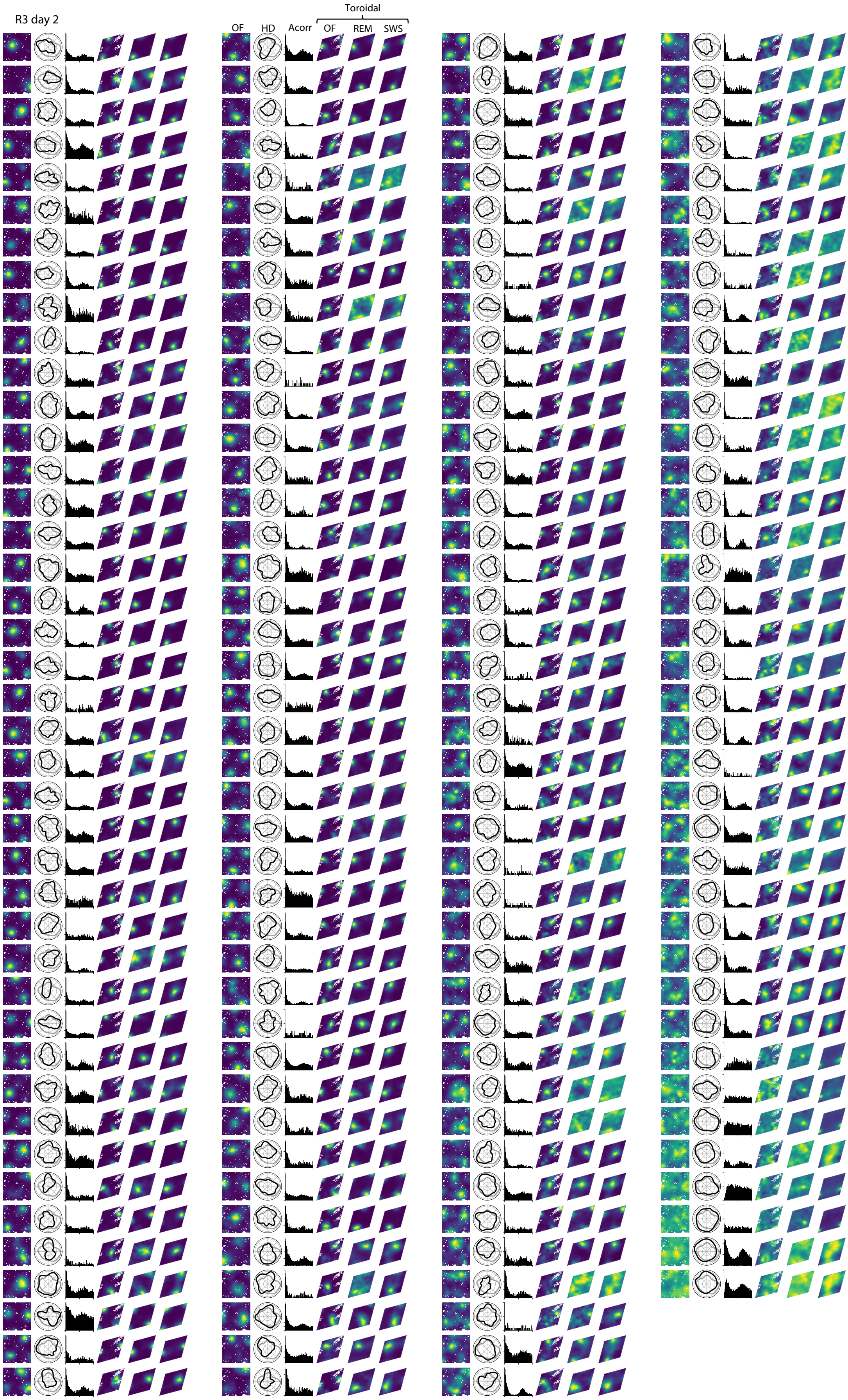
R3 day 2
Toroidal
OF
HD
Acorr
OF
REM
SWS

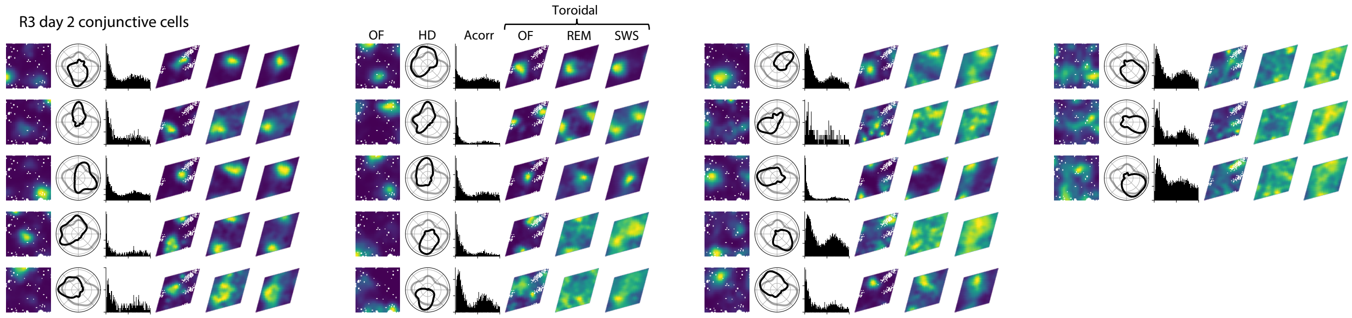

R3 day 2 conjunctive cells
Toroidal
OF
HD
Acorr
OF
REM
SWS

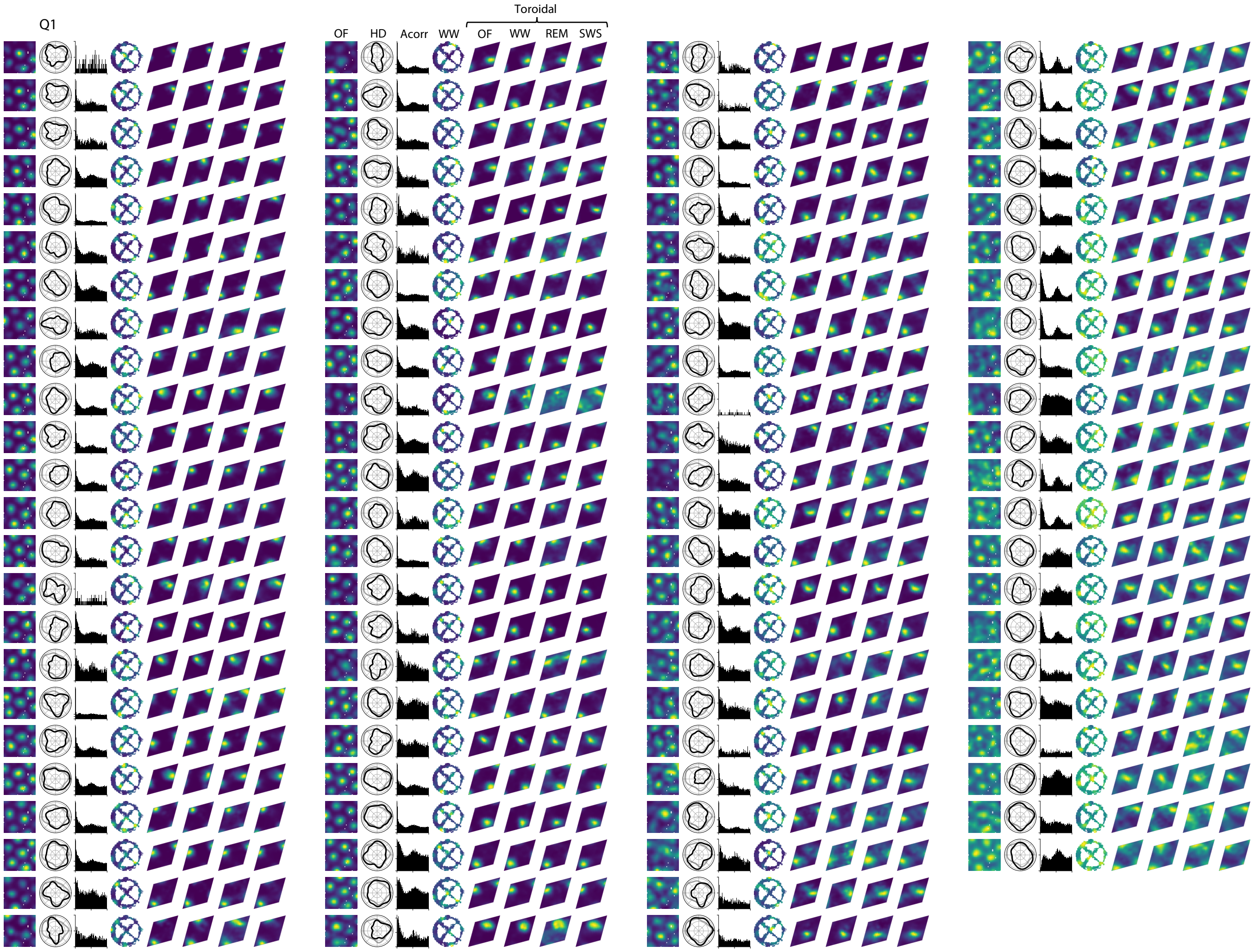

Q1
OF
HD
Acorr
WW
Toroidal
OF
WW
REM
SWS

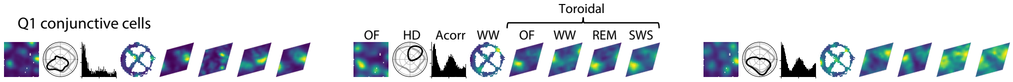

Q1 conjunctive cells
OF
HD
Acorr
WW
Toroidal
OF
WW
REM
SWS

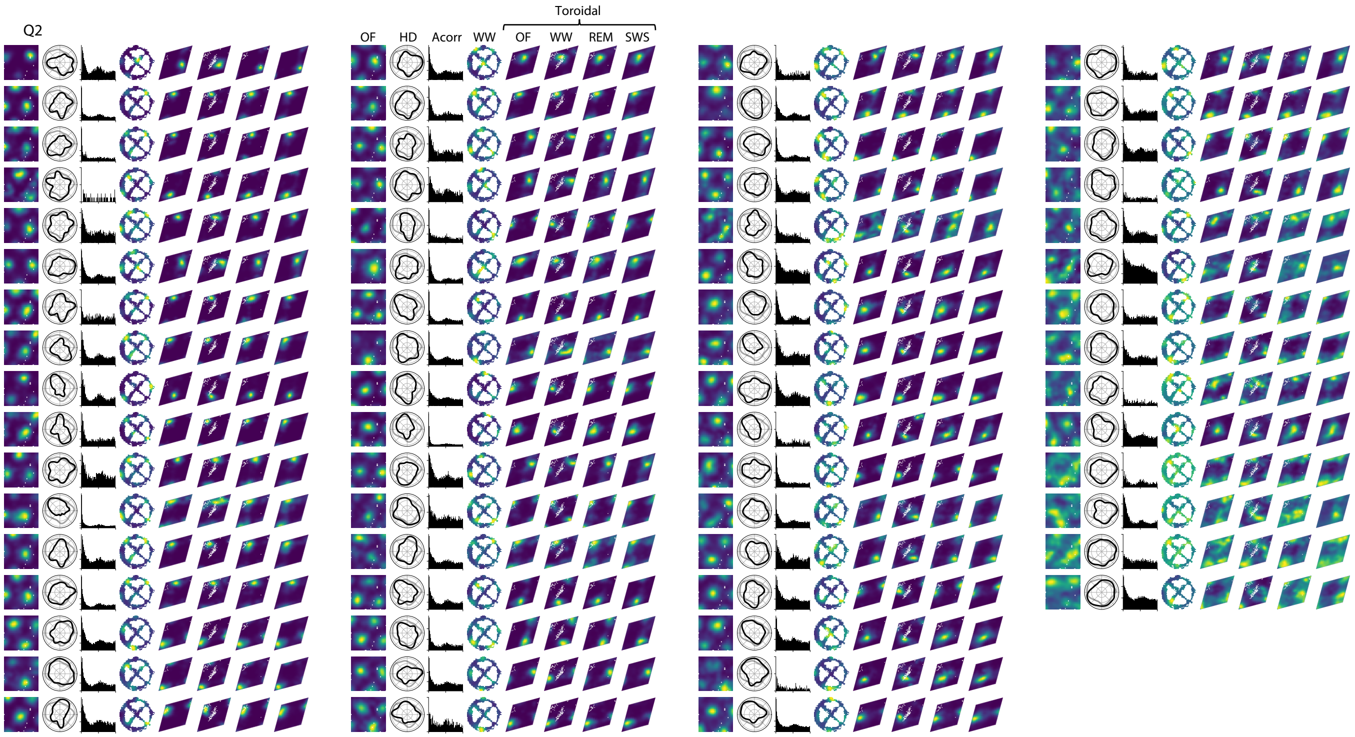
Q2
Toroidal
OF
HD
Acorr
WW
OF
WW
REM
SWS

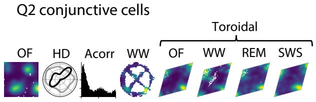
Q2 conjunctive cells
Toroidal
OF
HD
Acorr
WW
OF
WW
REM
SWS

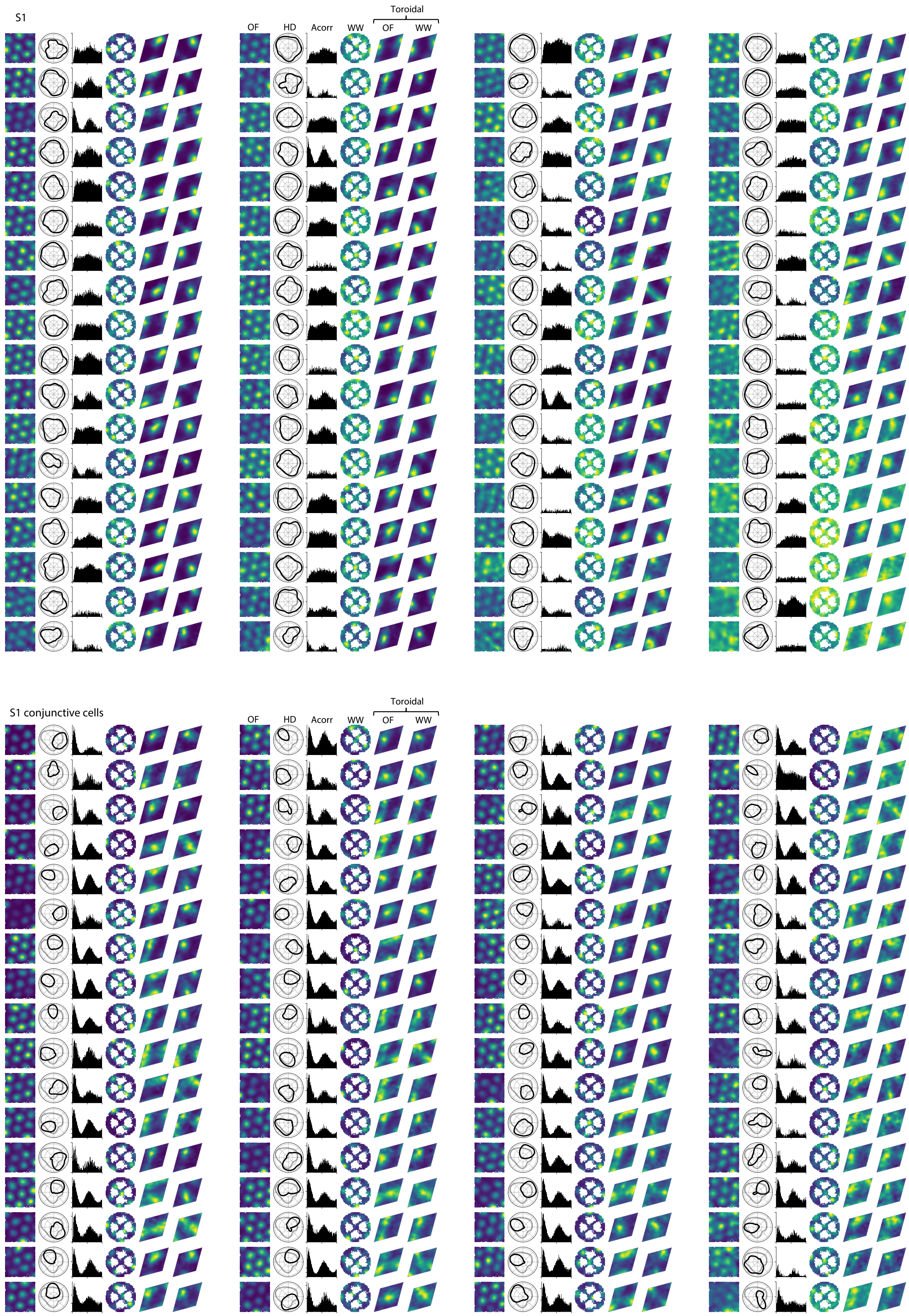
S1
OF
HD
Acorr
WW
Toroidal
OF
WW
S1 conjunctive cells
OF
HD
Acorr
WW
Toroidal
OF
WW

Supplementary Methods

Theoretical explanation of the six-dimensionality proposed by PCA

To understand why a minimum of six PCA components is necessary in order to account for a large fraction of the variance in the population patterns of grid cells, we shall consider an idealized model of grid cell firing, with the following three assumptions:

First, grid cell population activity patterns lie on a two-dimensional manifold with toroidal topology. This 2-D surface can be mapped to a rhombus with an angle of 60 degrees and periodic boundaries. Specifically, we can parametrize positions on the rhombus in the form $\alpha_1\, \vec{u}_1 + \alpha_2 \vec{u}_2$, where $\vec{u}_1$, and $\vec{u}_2$ are unit vectors whose orientations differ by 60 degrees, and $\alpha_{1,2} \in [0,1]$.

Second, the tuning of an individual grid cell to the toroidal coordinates (the position on the rhombus) is identical in all cells, up to a translation:

$$r_i = \phi\left(\left[\vec{\theta} - \vec{\theta}_i\right]_p\right)$$

where $\vec{\theta}$ is the 2-D position on the rhombus, $\vec{\theta}_i$ is the center of the receptive field of cell i, $\left[\vec{\theta} - \vec{\theta}_i\right]_p$ represents a shift of $\vec{\theta}$ by $\vec{\theta}_i$ with periodic boundary conditions on the rhombus and ϕ represents the structure of the tuning function.

Third, activity patterns of the neural population uniformly sample the states that correspond to different positions on the rhombus.

Under these conditions, the covariance matrix C of neural activity has the structure

$$C_{ij} = c\left(\left[\vec{\theta}_i - \vec{\theta}_j\right]_p\right).$$

If we further assume that receptive field centers $\vec{\theta}_i$ uniformly and regularly sample the rhombus, the covariance matrix commutes with periodic rigid translation operators on the rhombus. A full basis of eigenvectors of C can then be obtained such that the eigenvectors are also eigenvectors of the translation operators. Thus, these eigenvectors are Fourier modes of the form

$$\psi_i\left[\vec{k}\right] = A \exp\left(i\, \vec{k} \cdot \vec{\theta}_i\right),$$

where the wavevector $\vec{k}$ must be selected such that ψ is periodic on the rhombus. To achieve this requirement, $\vec{k}$ must be a vector lying on a vertex of a triangular lattice: the reciprocal of the lattice with basis vectors $\vec{u}_1$, $\vec{u}_2$.

The eigenvalues of C are the corresponding Fourier transform components of c. Typically, for a unimodal tuning curve, these eigenvalues will be a monotonically decreasing function of $|\vec{k}|$ for $|\vec{k}| > 0$. Furthermore, if the tuning of individual cells is isotropic, the eigenvalues depend only on $|\vec{k}|$. Therefore, the six PCA modes that correspond to the smallest value of $|\vec{k}|$ must contribute equally to the variance. If the tuning curve of individual cells is sufficiently wide, the eigenvalues are expected to decay rapidly with $|\vec{k}|$, and the modes that correspond to the first six PCA components are expected to capture a large fraction of the variance.

Theoretical background to persistent (co)homology

As persistent cohomology is a cornerstone of the current analyses, we wish to elaborate on its theoretical background to elucidate how and why it works (broader perspectives on topological data analysis and its application in biology are available elsewhere[57,58,93]). We start by introducing (co)homology and the Vietoris-Rips complex before we turn to persistent homology.

Given a topological space X, we can assign, for all natural numbers n, a vector space $H_i(X)$, called the i-th homology group of X, such that if $f: X \to Y$ is a continuous map between topological spaces X and Y, then $f_*: H_i(X) \to H_i(Y)$ is a linear map between vector spaces. Similarly, we may define cohomology groups, $H^i(X)$ by reversing arrows. These are dual notions and give the same results in our case. We will thus only continue describing the former. The dimension of $H_i(X)$ is called the i-th Betti number, representing the number of i-dimensional holes (for further details see Hatcher, 2002[60]). However, this may vary depending on the choice of coefficients of the vector space (note that it is choosing algebraic fields as coefficients that makes the homology groups vector spaces). For example, using $\mathbb{Z}_2$-coefficients, the Klein bottle will have the same homology as a torus. To separate these, we use $\mathbb{Z}_{47}$-coefficients in our computations. The choice of field coefficients simplifies computations at the risk of losing topological information known as *torsion*, measuring the

orientability of a space. However, the same number of holes (Betti numbers) is computed, which is what is here used to distinguish spaces.

As a point cloud is finite and discrete, its homology only returns the number of points in the point cloud (its 0-th Betti number). Thus, we associate combinatorial spaces known as simplicial complexes to the point cloud which may have non-trivial topology reflecting interesting structure and information of the data set and whose homology is easy to compute. A simplicial complex is a set V of vertices and a set S of finite non-empty subsets of V called simplices such that any vertex is a simplex and any non-empty subset of a simplex is a simplex. A simplex of cardinality $p + 1$ is referred to as a p-simplex (of simplicial dimension p) and geometrically, we may refer to a 0-simplex as a point, a 1-simplex as an edge, a 2-simplex a triangle, a 3-simplex a tetrahedron and so on in higher dimensions.

There are different choices in constructing simplicial complexes associated with the data. We used what is known as the Vietoris-Rips complex, here denoted R_r. The vertices of the Vietoris-Rips complex are the points in the point cloud and the simplices are the sets of points whose pairwise distance is less than the scale value, r. This is equivalent to replacing each point by a ball of common radius r and connecting two points with an edge if their balls intersect. p-simplices are then formed if each point of a subset of $p + 1$ points have edges to all other points in the subset (i.e. a $p + 1$-clique). Although this simplicial complex is not homotopy equivalent to taking the union of balls (as e.g. the Čech complex is) and thus does not necessarily have the same homology, the basic topological information is preserved under this correspondence.

One way to construct the Vietoris-Rips complex in detecting the topology of neural data is to regard individual cells as points and their pairwise dissimilarity (e.g. correlation) as scale. In our case, we rather considered the population activity vectors as the points. There is a subtle correspondence between these constructions, where the resulting barcodes are the same when applying persistent cohomology. This relationship is only valid when the tuning of the cells is such that the response is convex, seemingly invalidated by the firing patterns of grid cells with respect to its physical position in the environment. However, when considering the tuning to be a function of the toroidal state space, we find it indeed to be convex (indicated

by the single bumps in the toroidal rate maps for each grid cell – Fig 3a, Extended Data Fig. 9)[94], suggesting both constructions should give rise to the same barcodes.

We consider the nested chain of Vietoris-Rips complexes, R, constructed for all increasing values of r in which new simplices are formed:

$$R_{r_0} \subset R_{r_1} \subset \cdots \subset R_{r_n},$$

where $r_0 = 0$ and r_n is the largest pairwise distance in the point cloud, and apply homology to get a sequence of vector spaces and maps, for all dimensions i:

$$H_i(R_{r_0}) \to H_i(R_{r_1}) \to \cdots \to H_i(R_{r_n}),$$

where the maps are induced by the inclusion maps (note that we have omitted compositions and identity maps), called the i-th persistent homology. This may again be decomposed into a sum of elementary *persistence modules*[95]:

$$H_i(R) \cong \bigoplus_k I([b_k, d_k)),$$

where $b_k < d_k$ give the scales in which a class in $H_i(R)$ first appears and later disappears. Thus, we may represent the persistent (co)homology by displaying the intervals as bars starting at b_k and ending at d_k. The collection of bars for all dimensions results in what is known as the *barcode*.

The barcodes are shown to be stable under influence of noise (given certain assumptions on the construction)[96]. This means that small perturbations to the point cloud lead to small changes in the barcode. Thus, we note that in dimensionality reduction, quantifying the dissimilarity between the barcode of the high-dimensional representation and its embedding may address the challenge of measuring how faithful an embedding is[53].

Supplementary References

93. Rabadan, R. & Blumberg, A. J. *Topological Data Analysis for Genomics and Evolution: Topology in Biology*. (Cambridge University Press, 2019). doi:10.1017/9781316671665.

94. Curto, C. What can topology tell us about the neural code? *Bull. Am. Math. Soc.* **54**, 63–78 (2017).

95. Chazal, F., De Silva, V., Glisse, M. & Oudot, S. *The structure and stability of persistence modules*. (Springer, 2016).

96. Cohen-Steiner, D., Edelsbrunner, H. & Harer, J. Stability of persistence diagrams. *Discrete Comput. Geom.* **37**, 103–120 (2007).
